# Riolan arch pseudoaneurysm hemorrhage after endovascular covered stent-graft treatment of an abdominal aortic aneurysm: A case report

**DOI:** 10.1097/MD.0000000000017789

**Published:** 2019-11-27

**Authors:** Xunqiang Liu, Jinhui Zhang, Huanjun Chen, Liqiong Zhang, Hongtao Wang, Min Ji, Jifeng Wang, Enshuai Zhu, Jiaping Wang, Min Tian

**Affiliations:** a Department of Vascular Intervention; b Department of Ultrasound; c Department of Anesthesiology, Yan’an Affiliated Hospital of Kunming Medical University, Yunnan, China; d Department of Intervention, The Second Affiliated Hospital of Kunming Medical University, Kunming, China.

**Keywords:** covered stent-graft, coil embolization, pseudoaneurysm, Riolan arch

## Abstract

**Introduction:**

Riolan arch thickening is usually caused by the occlusion of the superior mesenteric artery (SMA), inferior mesenteric artery, or abdominal aortic artery, by colon cancer, or by ulcerative colitis in the active phase.

**Patient concerns:**

A 61-years-old female was admitted due to left lower abdominal pain, nausea, and vomiting for more than 4 days. She had received an endovascular covered stent-graft exclusion due to abdominal aortic aneurysm 18 months earlier. Computed tomographic angiography (CTA) showed a local rupture of 1 of the branch artery of the SMA, and a pseudoaneurysm was formed around it. It was feared that performing Riolan atrial arch pseudoaneurysm embolization may cause ischemia of the inferior mesenteric artery (IMA) and could lead to avascular necrosis of the descending colon and sigmoid colon, intestinal perforation, and peritonitis.

**Diagnosis:**

Riolan arch collateral circulation associated with pseudoaneurysm hemorrhage after endovascular covered stent-graft treatment of an abdominal aortic aneurysm.

**Interventions:**

Riolan arterial arch pseudoaneurysm embolization was performed near the distal end.

**Outcomes:**

The symptoms, signs, and biochemistry returned to normal.

**Conclusion:**

Riolan arch collateral circulation can be caused by pseudoaneurysm hemorrhage after endovascular covered stent-graft treatment of an abdominal aortic aneurysm.

## 1 Introduction

The Riolan arterial arch was proposed by the French anatomist Jean Riolan in 1965. It is formed by the anastomosis of the superior mesenteric artery (SMA), the middle colic artery, and the inferior mesenteric artery (IMA). Alternatively, it can be formed by the anastomosis of other branch arteries of the SMA and the ascending branch of the left colic artery. It is an important collateral artery connecting the SMA and IMA, and is located on the inner side and run along the root of the mesentery. The Riolan arterial arch usually has a small blood flow and slow flow rate, and is not easily displayed clearly during angiography.^[1]^

The Riolan arch is absent in 1% of the population.^[2]^ The display of the Riolan arch on angiography is usually due to stenosis or gradual occlusion of the SMA, IMA, and abdominal aortic artery, leading to chronic intestinal ischemia and the opening of collateral circulation for compensation.^[3]^ The Riolan arterial arch is then distorted and dilated, and the vessel gradually thickens, for a diameter of 2.50 to 5.27 mm (mean of 3.83 ± 0.60 mm).^[4]^ With this collateral, blood flow gradually increases and the intestinal ischemia is alleviated. The collateral circulation can also be caused by colon cancer and ulcerative colitis in the active phase.^[5]^

We present 1 case of Riolan arch collateral circulation associated with pseudoaneurysm hemorrhage after endovascular covered stent-graft treatment of an abdominal aortic aneurysm. Standard care is performed, so ethical approval is not applicable in this study. Written informed consent was obtained from the patient.

## 2 Case report

A 61-years-old female was admitted to the emergency department of “our hospital at 13:08 on September 10, 2018 due to left lower abdominal pain, nausea, and vomiting for more than 4 days. There was no apparent cause for the abdominal pain, which was persistent and progressively aggravated. She vomited twice (bile only at a 3-hour interval) and the abdominal pain was not relieved after vomiting. She was cold, sweating, and fatigued, but without shortness of breath, wheezing, or dyspnea. Body temperature was 36.5 °C, pulse was 102 beats/minutes, and blood pressure was 158/106 mm Hg. The patient had an anemic appearance, with pale skin and mucosa. The abdomen was flat and soft, tender in the lower left part, without rebound tenderness, without muscle tension, and without palpable mass. The shifting dullness was positive and normal bowel sounds could be heard. Hemoglobin was 69 g/L, white blood cells count (WBC) was 6.97 × 10^9^/L, neutrophil was 81.4%, albumin was 31 g/L, and blood natriuretic peptide (BNP) was 327 ng/L. Blood coagulation function was normal. Abdominal ultrasound showed a large amount of effusion in the abdominal cavity. Color ultrasound examination of the heart showed that the left ventricular diastolic function was decreased, with an ejection fraction (LVEF) of 60%. The patient had poor mental state and sleep since the onset of the symptoms, and extremely poor appetite. The patient was menopausal and had no history of hypertension or any other chronic diseases.

**Figure 1:**
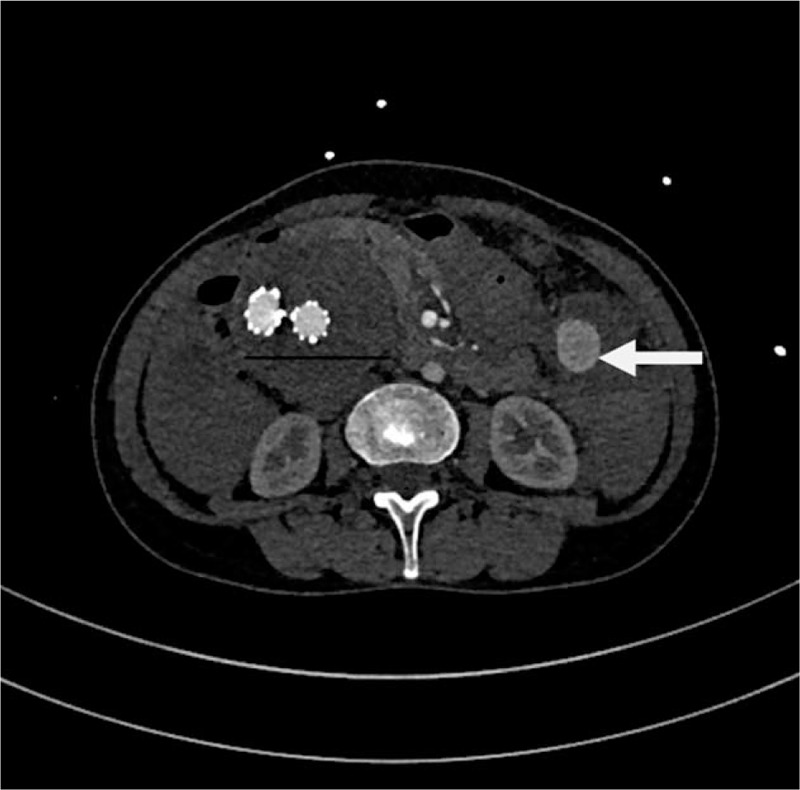
Preoperative computed tomography (CT) showing the aneurysm signs in the left abdomen, with effusion.

**Figure 2:**
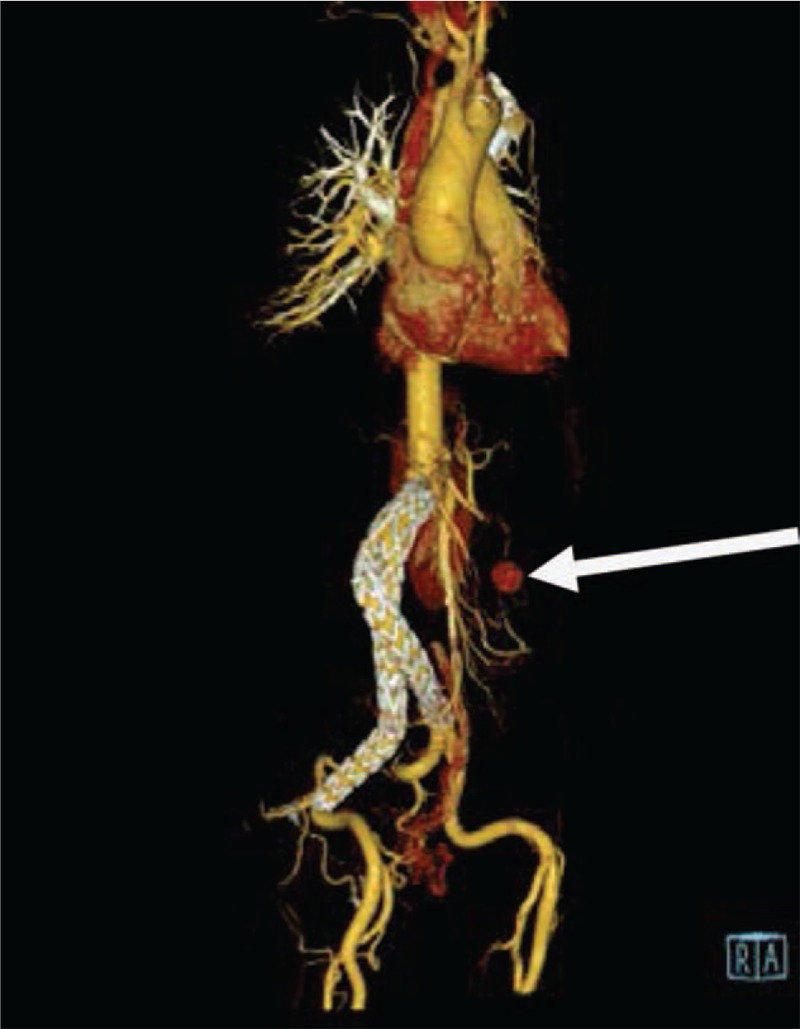
Preoperative aortic computed tomographic angiography (CTA) showing pseudoaneurysm signs in the distal end of a branch artery of superior mesenteric artery (SMA).

She had received an endovascular covered stent-graft exclusion due to abdominal aortic aneurysm at our hospital on June 1, 2016. The 4 covered stents used were from MicroPort Endovascular MedTech Co, Ltd (HBB2414-170-1500, 1612-120, 1612-80 and 1612-100). The bilateral iliac artery stent was to the distal part of the inferior common iliac artery and the bilateral internal iliac artery was retained. The patient did not take anticoagulants, nor did she undergo regular reexaminations. Computed tomographic angiography (CTA) showed a local rupture of one of the branch artery of the SMA, and a pseudoaneurysm was formed around it. A large amount of hematocele was observed in the abdominal cavity (Figs. 1 and 2). After stent implantation for abdominal iliac aneurysm, a thick mural thrombus was formed around the stent (Fig. 1).

The patient was then admitted to the department of vascular interventional therapy. The diagnoses were:

1. SMA branch artery pseudoaneurysm rupture hemorrhage;
2. abdominal hematocele;
3. hemorrhagic anemia; and
4. postoperative endovascular graft exclusion for abdominal aortic aneurysm.

**Figure 3:**
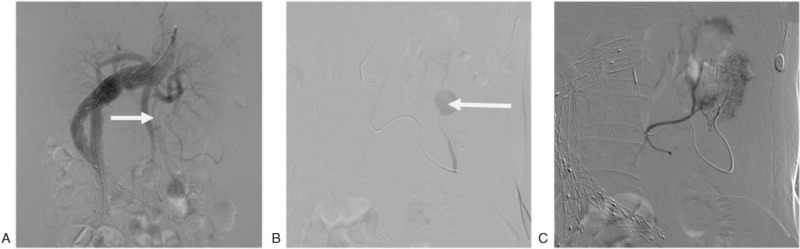
(A) Abdominal aortic angiography: a branch artery at the initial segment of the superior mesenteric artery (SMA) was distorted and thickened. (B) A pseudoaneurysm formed in the Riolan arterial arch. (C) Angiography of the distal end of the pseudoaneurysm showed that the artery was connected to the ascending branch of the left colonic artery.

The patient underwent SMA angiography and SMA hemorrhage branch arterial embolization emergently. A 5-F sheath was placed through the right femoral artery approach. The abdominal aortic angiography showed that the celiac trunk artery, hepatic artery, splenic artery, and bilateral renal arteries were well developed, and no contrast agent spilled out (iodixanol, 16 g in 50 ml, Yangzijiang Pharmaceutical Group Co., Taizhou, Jiangsu, China). The SMA trunk and branch arteries were well developed and no contrast agent spilled out. At the initial segment, a branch of the vessel was twisted, thickened, and extended to the left abdomen, and there was a sign of pseudoaneurysm at the distal end (Fig. 3). The IMA trunk and branch arteries were not developed, but the stent in the abdominal iliac artery was well developed, and no inner leak was observed. It was difficult to arrive at the SMA using the Yashiro catheter and the super-selective Cobra catheter had to be used because the abdominal aorta was distorted.

Therefore, the approach was changed to a 5-F atrial sheath with the left brachial artery approach. The Cordis VER single elbow catheter was inserted into the SMA for angiography to the proximal end of the SMA for indwelling. The Progreat microcatheter (Terumo, Japan) was inserted through the VER single elbow catheter, and the super-selective micro-guidewire was inserted to the site of the distortion and thickening and extended to the branch artery at the left abdomen for angiography. It showed that the vessel was distorted and thickened, with a diameter of about 2.9 mm. In the middle segment of the artery near the descending colon that turned to the splenic flexure of colon, a pseudoaneurysm with a diameter of about 2 cm could be observed, and the contrast agent spilled out. A vortex was observed in the aneurysm cavity. The distal artery of the pseudoaneurysm was normal in morphology. Angiography was performed by inserting a Progreat microcatheter into the artery at the distal segment of the pseudoaneurysm under the guidance of the micro-guidewire. It showed that the distal segment of the vessel communicated with the ascending branch of the left colonic artery; the sigmoid colon artery and the superior rectal artery were developed.

It was confirmed that the artery was the Riolan arterial arch between the SMA and the IMA. It was feared that performing Riolan atrial arch pseudoaneurysm embolization may cause ischemia of the IMA and could lead to avascular necrosis of the descending colon and sigmoid colon, intestinal perforation, and peritonitis. Therefore, it was recommended to directly perform exploratory laparotomy, Riolan atrial arch pseudoaneurysm resection, and Riolan atrial arch reconstruction.

**Figure 4:**
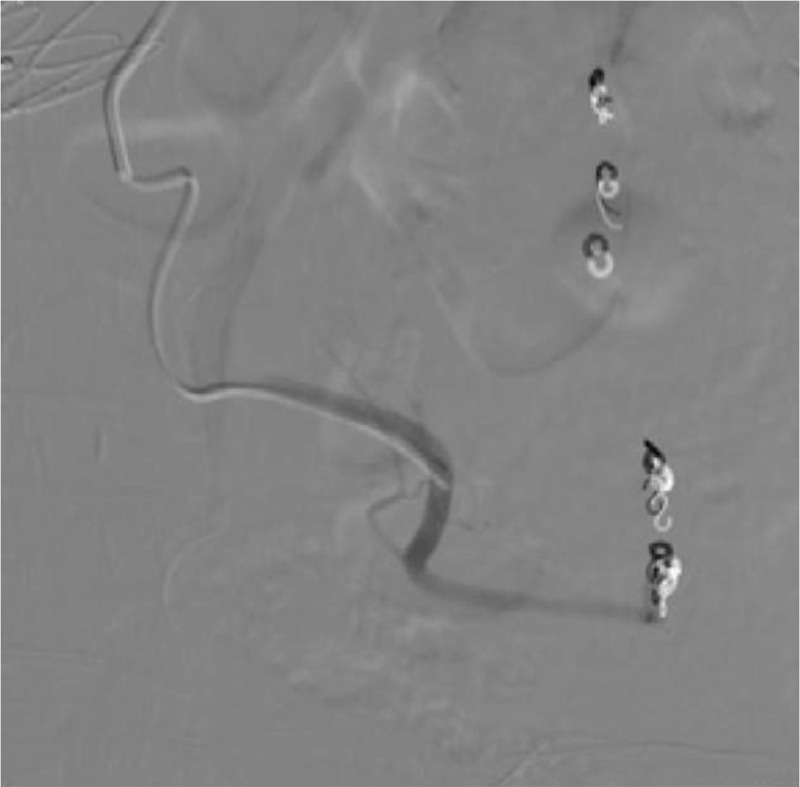
Angiography after embolization of the artery near the distal end of the pseudoaneurysm in the Riolan atrial arch showing that the coils were in good position. The sigmoid colon artery and superior rectal artery were developed and the pseudoaneurysm was not developed.

After explaining the disease and possible treatment to the patient and family, they refused Riolan atrial arch reconstruction. Since the Riolan arterial arch pseudoaneurysm was ruptured, with continuous hemorrhage and hemorrhagic shock, we decided to perform Riolan arterial arch pseudoaneurysm embolization near the distal end. Four pushable fiber-coated platinum coils (Boston Scientific, Natick, MA, USA, M0013812030) were inserted from the distal end of the Riolan arch through the microcatheter. One of the coils dropped into the aneurysm cavity, and 5 were inserted from the proximal end successfully (1 M0013812030, 3 M0013120210, and 1 M013812040). Repeat angiography after embolization showed that the artery in the distal end of the aneurysm and the pseudoaneurysm cavity were not developed. The blood flow at the proximal end was slow (Fig. 4).

Intraoperative bleeding was 5 ml at the puncture point. The patient was infused with 6 U of suspended red blood cells and 300 ml of fresh frozen plasma during and after surgery. The patient underwent fasting, gastrointestinal decompression, antibiotic (cefoxitin sodium, 1.0 g, 2.0 g, im, bid), nutrition (20% medium/long chain fat emulsion injection 250 ml, Guangzhou Qiaoguang), and other supportive treatments after surgery.

Abdominal pain, abdominal distension, and bowel sounds were closely observed. The abdominal pain and distension were gradually relieved after surgery. The borborygmus was 4/minute, the stool was yellow, without blood or black color. The diet was gradually switched from fluid to semi-liquid to general food.

**Figure 5:**
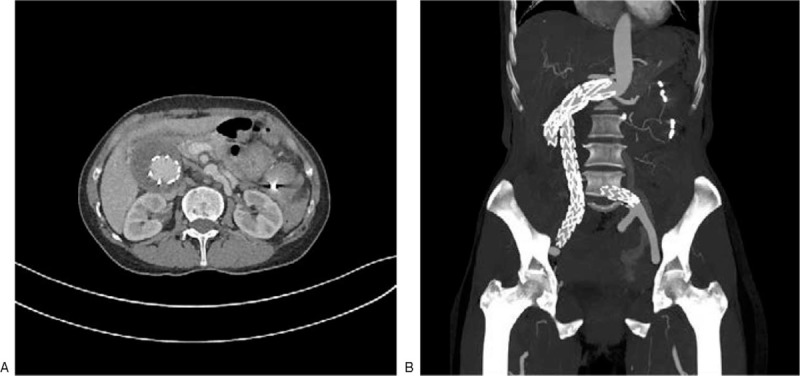
(A) Postoperative computed tomography (CT) reexamination showed that the pseudoaneurysm was not developed. (B) Postoperative aortic computed tomographic angiography (CTA) showing that the pseudoaneurysm was not developed.

**Figure 6:**
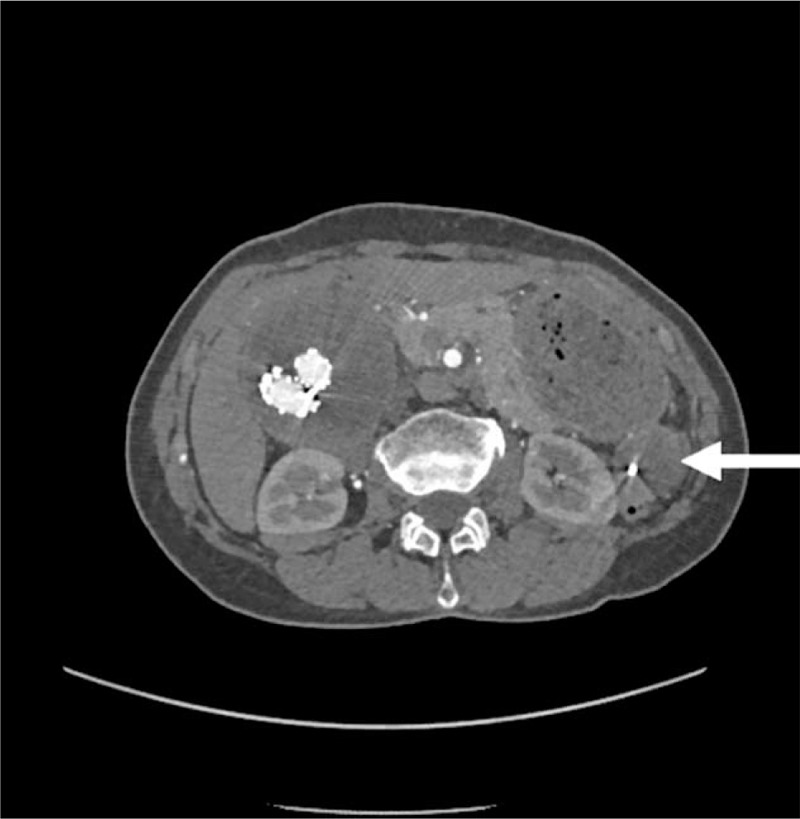
Contrast-enhanced computed tomography (CT) 2 months after surgery. There was thrombosis in the pseudoaneurysm. High-density metal shadow was seen in and around the pseudoaneurysm. There was no effusion in the abdominal and pelvic cavity.

On the 7th day after surgery, there was no abdominal pain and distension, and the bowel sounds were normal. Abdominal enhanced CT showed that there was no edema of the intestinal wall, no intestinal obstruction signs, and no hematocele in the abdominal pelvic cavity (Fig. 5). Abdominal ultrasound showed that the abdominal pelvic effusion was decreased compared with before surgery. Hemoglobin was 109 g/L, WBC was 5.05 × 10^9^/L, neutrophils were 70.6%, albumin was 37.4 g/L, and BNP was 49 ng/L. The patient was discharged on September 19, 2018 (on the 9th day after surgery). Follow-up at 2 months showed that the patient had no abdominal pain, distension, and no bloody and black stools. The abdomen was soft, without tenderness, rebound tenderness, or mass. Borborygmus was 5 times/minutes. Hemoglobin was 137 g/L, WBC was 4.95 × 10^9^/L, neutrophils were 58.62%, and albumin was 46 g/L. Abdominal ultrasound showed no effusion in the pelvic cavity. Abdominal enhanced CT showed left atrial pseudoaneurysm thrombosis, high density metal shadow in the cavity and around, no effusion in the abdominal cavity (Fig. 6).

## 3 Discussion

**Figure 7:**
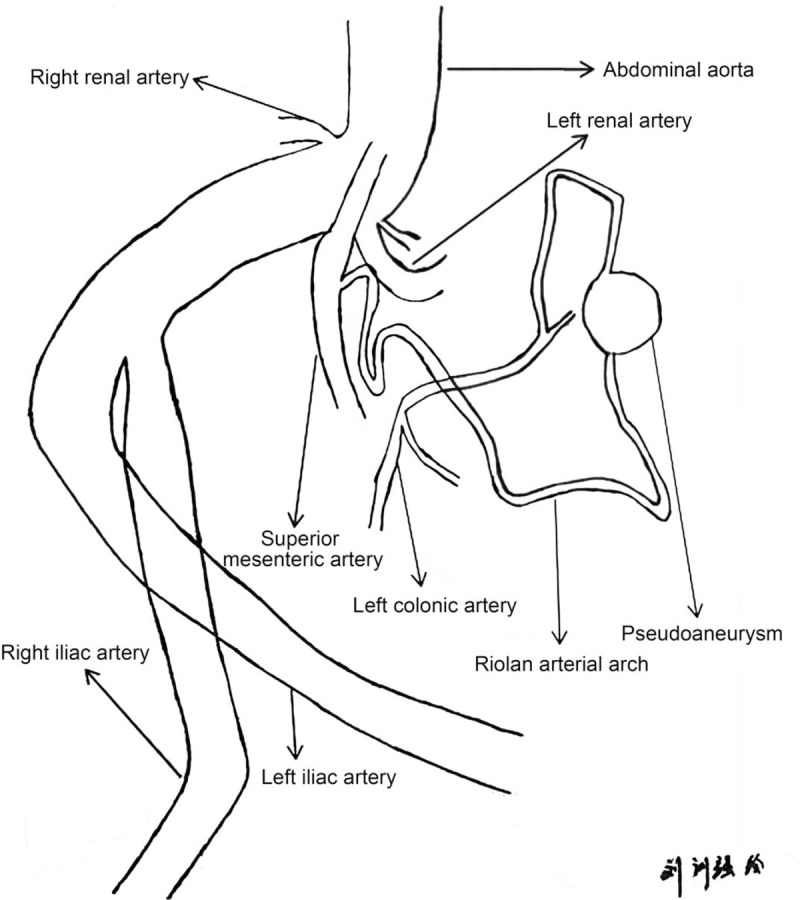
The Riolan arterial arch of the patient.

The patient underwent endovascular covered stent-graft exclusion for abdominal aortic aneurysm about 18 months before admission. The IMA root was isolated during the surgery. The blood supply to the descending colon and sigmoid colon depended on the compensation of collateral circulation opening. The branch artery at the initial segment of SMA was anastomosed with the ascending branch of the left colic artery to form the Riolan atrial arch. At this time, the blood supply to the descending colon and sigmoid colon was mostly dependent on the gradually thickened Riolan arch (Fig. 7). The abdominal pain of the patient was due to the rupture and hemorrhage of a pseudoaneurysm in the Riolan atrial arch between the SMA and IMA. The preoperative abdominal iliac artery CTA suggested that the rupture hemorrhage was on a branch of the SMA. If performing Riolan atrial arch pseudoaneurysm embolization, ischemia of the IMA branch artery could occur, leading to avascular necrosis of the descending and sigmoid colon, intestinal perforation, and peritonitis. We recommended to perform exploratory laparotomy, Riolan atrial arch pseudoaneurysm resection, and Riolan atrial arch reconstruction. It was thought superior to interventional embolization surgery. Unfortunately, hemorrhagic shock occurred and the family members refused the open surgery. Then, Riolan atrial arch pseudoaneurysm embolization and hemostasis was performed. In order to prevent the avascular necrosis of the descending and sigmoid colon, the patent underwent fasting, gastrointestinal decompression, nutrition, and supportive treatment. The abdominal pain, abdominal distension, and bowel sounds returned to normal after embolization. There was no intestinal ischemia or intestinal necrosis.

By analyzing the anatomy, other collateral arteries, in addition to the Riolan arterial arch, could act as collateral circulation between the SMA and IMA:

1. the Drummond marginal artery, also known as the marginal arterial arch, originating from the IMA and reaching to the left branch of the middle colonic artery;^[6]^
2. the ascending branch of the left colonic artery anastomosed with the left branch of the middle colonic artery; and
3. anastomoses between the superior rectal artery and the inferior rectal artery branched from the anterior trunk of internal iliac artery.

The bilateral internal iliac arteries were preserved in the abdominal aortic endovascular graft exclusion in this patient. It did not affect the blood supply of the inferior rectal artery. In this patient, the blood supply to the descending colon and sigmoid colon was mainly dependent on the Drummond marginal artery, as well as the anastomoses between the superior and inferior rectal arteries, after Riolan atrial arch pseudoaneurysm embolization. It was precisely because of these rich anastomotic arteries that the blood supply to the descending colon and sigmoid colon was not greatly affected, so that there was no avascular necrosis of sigmoid colon.

Nevertheless, although there were many anastomoses between the SMA and IMA, sometimes the anastomoses are poor or there was an interruption, so there is still weakness in the marginal arteries.^[7]^ Performing exploratory laparotomy, Riolan arterial arch pseudoaneurysm resection, and broken end vascular reconstruction can avoid avascular necrosis of the descending colon and sigmoid colon. Importantly, there was a need for gentle operation and careful surgery, avoiding damage to the Drummond marginal artery as much as possible. If the Drummond marginal artery was damaged during surgery, avascular necrosis of the descending, and sigmoid colon was highly possible.

Although such a case was rare, its treatment was in line with a previous study of abdominal pseudoaneurysms.^[8]^ A study showed that endovascular stent-assisted treatment of SMA occlusion was safe and effective, with good outcomes,^[7]^ supporting the treatment approach selected in the case reported here. A case of spontaneous SMA pseudoaneurysm has been reported, which was treated in a similar fashion as the present case, also with good outcomes, indicating that endovascular coil embolisation was a safe and effective technique.^[9]^ Finally, a case of traumatic IMA pseudoaneurysm has been reported, which was also treated with successful coil embolization.^[10]^

## 4 Conclusion

For this patient, the preoperative diagnosis was rupture hemorrhage of a pseudoaneurysm in the branch artery of the SMA. Riolan arch collateral circulation can be caused by pseudoaneurysm hemorrhage after endovascular covered stent-graft treatment of an abdominal aortic aneurysm. The therapeutic approach should be determined according to the specific characteristics of the disease and the wishes of the patients. This kind of medical condition is extremely rare, and more cases are needed to determine the optimal treatment and the long-term effects.

## Author contributions

**Conceptualization:** Xunqiang Liu, Jinhui Zhang, Min Tian.

**Data curation:** Xunqiang Liu, Jinhui Zhang, Huanjun Chen, Liqiong Zhang, Hongtao Wang, Min Ji, Jifeng Wang, Enshuai Zhu, Jiaping Wang.

**Formal analysis:** Xunqiang Liu, Jinhui Zhang, Huanjun Chen, Liqiong Zhang, Hongtao Wang, Min Ji, Jifeng Wang, Enshuai Zhu, Jiaping Wang, Min Tian.

**Funding acquisition:** Xunqiang Liu, Jinhui Zhang, Min Tian.

**Investigation:** Xunqiang Liu, Jinhui Zhang.

**Methodology:** Xunqiang Liu, Jinhui Zhang.

**Project administration:** Min Tian.

**Writing - original draft:** Xunqiang Liu, Jinhui Zhang.

**Writing - review & editing:** Huanjun Chen, Liqiong Zhang, Hongtao Wang, Min Ji, Jifeng Wang, Enshuai Zhu, Jiaping Wang, Min Tian.
